# Correspondence

**Published:** 1906-06

**Authors:** T. M. McIntosh

**Affiliations:** Thomasville, Ga.


### *CORRESPONDENCE.*

*Mr. Editor:*

I have just read your editorial in your May number, "An Ugly Fight," with much regret and even sorrow. Not regret and sorrow that you wrote it, but that conditions existed that could call forth such an editorial and justify it. This regret and sorrow is most felt because the American Medical Association, through its hired "walking delegates," as my friend, Dr. Hicks, calls him, but whom it would be just as appropriate to call a "paid lobbyist," has, during a session of the Medical Association of Georgia, been present, and under his hypnotic influence the chairman of the "Committee on Reorganization," Dr. McRea, sat silently by and allowed the Secretary, Dr. L. H. Jones, by a motion to take a draft of a Constitution, already submitted to the Association for future action, from its proper course and place it in the hands of the Executive Committee to report the next day at nine o'clock. To one who would think without favor, the conclusion would be that this was prearranged by Drs. McCormick, McRea and Jones to "railroad" the matter through, out of its usual and already prescribed course. And the "lobbyist" of the A. M. A., failing at this meeting to accomplish his purpose (and earn his salary paid by the great American Medical Association trust), presented himself at the next annual meeting, when a revolutionary and clearly unconstitutional proceeding, adopted a new Constitution inspired by the A. M. A.

Strange it is to me that the great body of the Medical Association of Georgia could have not seen the ulterior motive of the A. M. A. to create a gigantic and selfish medical oligarchy, a great financial and political body.

If the A. M. A. had been content to leave each State Association to manage its own affairs in its own way, without the intervention and presence of Dr. McCormick (the medical Andrew

Hamilton), it would not have concerned me so, but when, by his presence and his influence, he will be the cause of the disruption of the Medical Association of Georgia, of which I have been a member since my graduation, it is natural that I should have regret of such results, and be resentful toward him and not feel kindly toward those officers of the State Association who have aided him in his revolutionary work.

The Journal of the A. M. A., by reasons of its wealth, its wide circulation, is in a position to do a vast amount of original, pure, and abstract scientific work that would be of untold value to humanity and the medical profession, but the indications are that these powers will be used by the management to further their personal and financial schemes, and to crush already established legitimate enterprises and independent, private and unincorporated medical journals.

The characteristic and predominating impulses of a people are manifested in their deeds, and the signs which they display to the world.

The lusts that ruled the peoples of ancient days was shown in their Phallic emblems, their worship at the shrine of an Aphrodite, and their tribute to the court of Phryne, and from signs not hard to see, it may be that the Journal of the A. M. A. in its lust for undisputed power may become the harlot of American medical journalism, displaying its opulent charms of wealth and wide circulation to attract the patronage of any patent, proprietary or other advertisement that will pay the price.

Of the fight between the A. M. A. and the manufacturing chemist, I have no comments; it is their affair. Personally, I have never prescribed any preparations of whose constituents I am ignorant; so I do not prescribe either patent or proprietary preparations, as I do not know what they are. I am regretful of the whole affair. It was so unnecessary.

Respectfully,

T. M. MCINTOSH.

*Thomasville, Ga., May 16, 1906.*

